# Early Thrombocytopenia at Hospital Admission Predicts Mortality in Patients with Non-Isolated Severe Traumatic Brain Injury

**DOI:** 10.3390/biomedicines12122702

**Published:** 2024-11-26

**Authors:** Patricia Piñeiro, Alberto Calvo, María Dolores Pérez-Díaz, Silvia Ramos, Sergio García-Ramos, Mercedes Power, Isabel Solchaga, Cristina Rey, Javier Hortal, Fernando Turégano, Ignacio Garutti

**Affiliations:** 1 Department of Anesthesiology and Critical Care, Gregorio Marañón Universitary General Hospital, 28007 Madrid, Spain; bagullo76@hotmail.com (P.P.); silvia.ramos.cerro@gmail.com (S.R.); sergius_5@hotmail.com (S.G.-R.); poweresteban@gmail.com (M.P.); isolchaga91@gmail.com (I.S.); javier.hortal@gmail.com (J.H.); ngaruttimartinez@yahoo.es (I.G.); 2 Biomedical Research Foundation, Gregorio Maralón Universitary General Hospital, 28007 Madrid, Spain; 3 Department of General and Digestive Surgery, Gregorio Marañón Universitary General Hospital, 28007 Madrid, Spain; lolaperezdiaz@hotmail.com (M.D.P.-D.); c.reyvalcarcel@gmail.com (C.R.); fernando.turegano@salud.madrid.org (F.T.); 4 Department of Pharmacology, Medical School, Complutense University of Madrid, 28040 Madrid, Spain

**Keywords:** traumatic brain injury, intracranial hemorrhage, coagulopathy, thrombocytopenia, mortality, emergency department

## Abstract

Patients with severe traumatic brain injury (STBI) often experience an abnormal hemostasis that contributes to mortality and unfavorable neurological outcomes. Objectives: We aimed to analyze epidemiologic, clinical, and laboratory factors associated with mortality in patients with STBI during the first 48 h after in-hospital admission. Methods: We performed an observational retrospective study of STBI patients with associated extracranial trauma [defined as Injury Severity Score (ISS) ≥ 16 with an Abbreviated Injury Scale (AIS) head and neck ≥ 3 and Glasgow Coma Scale (GCS) ≤ 8] admitted to a Level II trauma center over seven years (2015–2021). Patients were divided into two groups: survivors and dead. We assessed differences regarding demographics, trauma severity, hemodynamics, disability, need for surgery, length of stay, transfusions, need for massive transfusion protocol, and hemostatic laboratory parameters at different time points. Results: A total of 134 STBI patients were included. Patients who died were older, mostly men, and showed higher trauma severity and disability. Hemoglobin, platelets, and clotting parameters deteriorated after admission to the emergency department (ED) with significant differences between groups within the first 24 h after admission. Platelet count < 150 × 10^3^/μL at ED arrival, GCS, and age were independent risk factors for mortality. Conclusions: Older age, GCS, and platelet count at ED arrival were independent risk factors for mortality in STBI patients with associated extracranial trauma. Early thrombocytopenia < 150 × 10^3^/μL at ED arrival may be used as a simple prognostic tool to early predict mortality between non-isolated STBI.

## 1. Introduction

Severe traumatic brain injury (STBI) is one of the main causes of death and disability throughout the world, yet associated neurological morbidity and mortality have not decreased significantly during the last 30 years [1].

The prognosis of patients with STBI is still uncertain, and multiple studies try to identify predictors of outcome in these patients. Patients with STBI often have intracranial bleeding, which can be accompanied by secondary injury. This secondary injury may consist of brain swelling and progression of hemorrhagic injury (PHI), which, ultimately, could form a risk of herniation and death [2]. In addition, STBI patients who suffer a traumatic intracranial hemorrhage remain at risk for developing a coagulopathy after trauma. After STBI, hemostasis is often derailed, leading to a hypocoagulopathic and—less often—hypercoagulopathic state [3] depending on injury pattern, presence of hypoperfusion, endothelial damage, hemostatic treatment, individual responses, genetic predisposition, and comorbidities [4].

The presence of coagulopathy is a well-recognized predictor of poor outcomes in patients with STBI, being associated with increased rates of disability and mortality due to the increased risk of PHI and brain edema progression [3,5,6]. 

Coagulopathy is usually defined as low platelet count or elevated international normalized ratio (INR), prothrombin time (PT), or activated partial thromboplastin time (APTT) [7]. However, the incidence and degree to which a specific coagulopathy of STBI contributes to the overall burden of TBI mortality remain unclear, although platelet count and dysfunction and coagulopathy appear to be relevant factors in isolated STBI [3,8,9]. In addition, STBI patients with associated extracranial trauma are difficult to diagnose and manage and may associate more hypotension and coagulopathy resulting from extracranial injuries, which further darkens the prognosis compared to isolated STBI [10]. On this basis, there is a lack of insight into STBI-associated coagulopathy that clearly contrasts with their potential clinical consequences.

Therefore, we aimed to study the influence of hemostatic abnormalities evaluated at the emergency department (ED) and hemostasis during the first 48 h after STBI to further determine whether dead, non-isolated STBI patients show a different hemostatic admission profile compared to non-isolated STBI survivors. Secondly, we also investigate whether other epidemiological and clinical characteristics were associated with an increased risk of mortality in these patients. In that case, STBI patients with associated extracranial trauma patients should be better characterized, and we could potentially implement earlier strategies to improve clinical outcomes.

## 2. Materials and Methods

We performed an observational descriptive retrospective study in a Level II equivalent trauma center (Gregorio Marañón Universitary General Hospital, Madrid, Spain) between 2015–2021. This study follows [11]. The study was approved by the research ethical committee of our institution (Drug Research Ethics Committee of Gregorio Marañón Universitary General Hospital). All research was performed in accordance with Strengthening the Reporting of Observational Studies in Epidemiology (STROBE) guidelines for retrospective cohort studies and with the Declaration of Helsinki. Due to its retrospective nature, informed consent was waived by our research ethical committee regulations.

Patients were recruited from our local trauma registry. Inclusion criteria were patients with non-isolated, severe TBI [defined as Injury Severity Score (ISS) ≥ 16 with an Abbreviated Injury Scale (AIS) head and neck ≥ 3 and Glasgow Coma Scale (GCS) ≤ 8], ≥ 18 years old, and admitted within 6 h of injury to our center. We excluded patients who were taking antiplatelet and/or anticoagulant medication; pregnant women; those with liver disease, chronic kidney disease on hemodialysis, chronic alcoholism, or previous hemostatic disease; and patients with diffuse axonal injury due to their possible role in hemostatic and neurological disturbances. 

**Figure 1:**
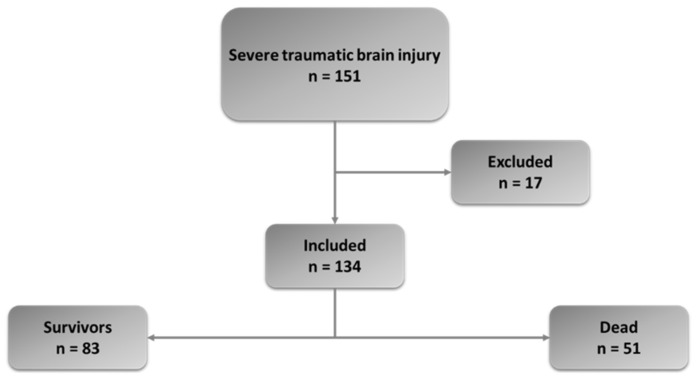
Flowchart of patients included in the study.

During the study period, a total of 151 STBI patients were admitted to our center. After excluding 17 patients, 134 patients composed the study group. Patients included were further analyzed in two subgroups: survivors (83) and dead (51) (Figure 1). 

### 2.1. Data Collection

All patients were initially admitted to the ED for initial assistance. Afterward, they were transferred to the radiology or operating room, depending on their clinical stability. On admission, all patients were clinically evaluated by a neurosurgeon and underwent a head computed tomography (CT) scan, except when extracranial surgery was necessary for immediate damage control, when the head CT scan was performed at the end of surgery. 

All evaluations of CT scans were extracted from the original report by the radiologist. Acute brain injuries assessed by CT imaging included the following: epidural hematoma (EDH), subdural hematoma (SDH), subarachnoid hemorrhage (SAH), intraparenchymal hemorrhage (IPH), and skull fracture. Volumes of EDH, SDH, and parenchymal/hemorrhagic contusions were measured. In addition, the status of the ambient cisterns and the presence of midline shift were assessed. The indication for neurosurgery was established by the neurosurgery staff in accordance with the recommendations established by the international guidelines for the surgical management of TBI [12]. In general, the surgical criteria were based on the level of consciousness, neurological focality, hematoma volume and/or thickness, presence of signs of cisternal compression, deviation of the midline > 5 mm, or maintained (>1 h) refractory intracranial pressure (>25 mmHg). After radiological study and/or surgery, patients were admitted to the surgical intensive care unit (SICU). The cause of death was obtained from the death report on the chart. Brain death was defined as irreversible cessation of vital activity of the whole brain (including the brain stem), verified by well-defined neurological clinical protocols and specialized tests (radiographic test and/or electroencephalogram) following international guidelines [13]. In addition, devastating brain injury was defined as any neurological condition assessed at the time of hospital admission, which represented an immediate threat to life or incompatible with good functional recovery, and where early limitation or withdrawal of therapy was considered [14]. 

Different epidemiological and clinical data were compared between groups: age, sex, time to hospital admission, cause of accident, pre-hospital fluid replacement, hemodynamics (heart rate and arterial blood pressure), neurologic parameters [GCS at admission, Glasgow Outcome Score (GOS) at discharge], trauma severity (ISS, AIS, AIS head and neck), length of stay in SICU, red blood cells (RBC) transfusions, need for massive transfusion protocol (MTP), need for urgent surgery and neurosurgery, and associated extracranial injury. MTP was activated when the Assessment of Blood Consumption (ABC) score was ≥2.

Laboratory blood tests were collected by arterial samples at different standardized times as part of our routine patient care: arrival to ED, admission to SICU, 24 h after SICU admission, and 48 h after SICU admission. Blood samples were taken to determine INR, PT and APTT, as well as values for fibrinogen, hemoglobin, and platelets, on arrival to the ED, admission to the SICU, and at 24 h and 48 h after admission to the SICU. Thrombocytopenia was defined as a platelet count < 150 × 10^3^/μL based on most extended threshold in the literature [15]. 

### 2.2. Data Availability

All data generated or analyzed during this study are included in this published article. The database is available from the corresponding author.

### 2.3. Statistical Analysis

Statistical analysis was performed using the IBM SPSS software package (Chicago, IL, USA, version 21.0). Categorical data are expressed as percentages. Quantitative data are shown as mean (standard deviation) for normally distributed data, while non-normally distributed data are expressed as median (interquartile range). We made a univariate analysis of mortality during hospital stay. Medians for numerical variables were compared between groups for continuous data using the Mann–Whitney test and χ^2^ test or Fisher’s exact test (when appropriate) to compare categorical variables. We then performed a logistic regression analysis for mortality, including variables, which had a *p* value < 0.1 in univariate analysis. Finally, for survival analysis according to platelet count < or >150 × 10^3^/μL, the Kaplan–Meier method was used, and the curves were compared using the log-rank test. Statistical significance was set at 0.05 *p*-value.

## 3. Results

During the observation period (2015–2021), a total of 151 non-isolated STBI patients were admitted to our center; 134 patients composed the study group. Out of these 134 patients, 83 (62%) survived, and 51 (38%) died. Regarding those who deceased, 45 (88%) died from neurological causes (brain death or devastating brain injury), and only 6 patients died from non-neurological causes, such as hemorrhagic shock or limitation of therapy. A total of 50% of patients died within 48 h of admission to hospital. 

### 3.1. Demographic and Clinical Characteristics of STBI

Patients who died were significantly older, male sex, and experienced less motorcycle collisions (15.6% vs. 24.1%, *p* = 0.011). They also significantly showed higher ISS (41 vs. 34, *p* = 0.003) and AIS head and neck (5 vs. 4, *p* < 0.001), while GCS (5 vs. 4, *p* < 0.001), GOS (1 vs. 3, *p* < 0.001), need for urgent surgery (35% vs. 70%, *p* < 0.001), and length of stay in SICU (2 vs. 17, *p* < 0.001) were lower compared to STBI survivors. No statistical differences between both groups were observed regarding the time to reach hospital, pre-hospital fluid replacement, other mechanisms of accidents, hemodynamics (systolic arterial pressure and heart rate), AIS overall, massive transfusion protocol activation, and RBC transfusions (Table 1).

### 3.2. Radiological Findings of the Initial CT

According to the radiological findings of the initial CT scan performed on admission (Table 2), we observed that in the dead group, most patients sustained traumatic SAH (58.8%) and SDH (56.9%), followed by cerebral contusion (27.5%) and IPH (23.5%), while in the survivors’ group, most patients experienced traumatic SAH (56%), followed by SDH (41.7%) and cerebral contusion (35.7%). Skull fractures were also common in both groups (54.9% vs. 40.5%). However, significant differences were only found between both groups in terms of IPH (23.5% vs. 2.4%, *p* < 0.001), cerebral contusion (23.5% vs. 35.7%, *p* = 0.03), and radiological signs of brain herniation (23.5% vs. 7.1, *p* = 0.007). No statistical differences were observed regarding the need for urgent neurosurgery between both groups (23.5% vs. 28.9%).

### 3.3. Associated Extracranial Injuries

In our sample, the most frequent associated extracranial injuries in both groups (Table 3) were those affecting the thorax (66.4%) and skeleton (65.6%), followed in order of frequency by the spinal column (47%), abdomen (34.3%), pelvis (31.3%), face (30.6%), retroperitoneal space (29.7%), and liver (28.2%). Extracranial injuries were equally distributed between STBI patients who survived and STBI patients who died, except for cervical spinal cord (50.6% vs. 41.2%), thorax (72.2% vs. 56.8%), and liver (20.5% vs. 9.8%) injuries, which were more common in patients who survived. However, no statistical differences with respect to extracranial injuries were observed between survivors and non-survivors.

### 3.4. Laboratory Abnormalities in Hemostasis

In order to study the laboratory abnormalities in hemostasis after STBI, we analyze differences between survivors and dead in regard to clotting values and platelet count during the first 48 h (Table 4). Within the first 24 h, INR and APTT deteriorated gradually in both groups, while PT increased in patients who survived and remained stable in those who died. After 48 h, INR, APTT, and PT returned to normal values. We only found significant differences regarding clotting parameters between survivors and dead STBI patients at SICU admission time, when dead patients showed prolonged INR, APTT, and PT times compared to survivors (*p* < 0.05). In contrast, fibrinogen levels increased exponentially at 24 h after SICU admission in both groups, at which point there were significant differences between survivors and dead (517 vs. 424, *p* = 0.023). 

**Figure 2:**
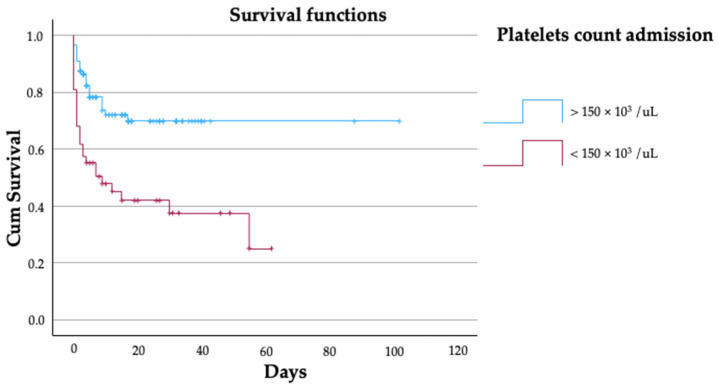
Kaplan–Meier survival curve for patients with a platelet count of > or <150 × 10^3^/μL. Kaplan–Meier survival curves are displayed to discriminate patients arriving at ED with >150 × 10^3^ platelets/μL (blue) and <150 × 10^3^ platelets/μL (red). The matched groups were compared using the log-rank test. Patients admitted with <150 × 10^3^ platelets/μL had significantly higher and earlier mortality than those admitted with >150 × 10^3^ platelets/μL (*p* < 0.001).

In addition, platelet count also decreased progressively after STBI in both groups, highlighting a marked initial lower platelet count recorded in patients who eventually died. Interestingly, we found that while platelet levels remained stable among patients who survived and only began to fall after 24 h, in patients who died, platelet levels decreased since ED arrival. In fact, platelet count was significantly lower in dead STBI patients when compared to survivors at all measured times (*p* < 0.001). Hemoglobin levels decreased clearly in both groups, with statistically significant lower values in dead STBI patients at ED arrival (*p* = 0.01) and SICU admission (*p* = 0.001). Furthermore, when we performed the Kaplan–Meier survival curve (Figure 2) for admitted patients with a platelet count < 150 × 10^3^/μL, we observed that ED-admitted patients with <150 × 10^3^/μL had a significant early drop in survival compared to those admitted with >150 × 10^3^/μL to the ED (*p* < 0.01), although both curves stabilized during the first few days after STBI.

### 3.5. Logistic Regression Analysis for Mortality

Finally, the logistic regression analysis (Table 5) revealed that age, GCS, and platelet count at ED arrival were independently associated with an increase in mortality.

## 4. Discussion

Our study investigating a sample of STBI patients with associated extracranial trauma over seven years showed the predictive value of blood routine tests at admission and clinical characteristics for mortality outcomes, highlighting the usefulness of early assessment of platelet count and function and the need to develop treatment strategies for coagulation abnormalities in non-isolated STBI patients.

In terms of clinical characteristics, STBI mortality is highly influenced by epidemiological and clinical characteristics [16,17]. In our cohort, patients who died were older and mostly male sex. Remarkably, the logistic regression model pointed out age as an independent predictor of STBI mortality, in accordance with the literature [17,18]. This higher risk of mortality in older STBI patients might be explained by their poor cardiovascular reserve in response to the stress generated by trauma, attenuation of cerebral autoregulation mechanisms, less tolerance to cerebral ischemia, comorbidities, or greater postoperative complications in case of urgent surgery [19]. Likewise, males usually show higher mortality rates among the series [20] because the number of males included in TBI studies is higher than females due to their increased probability of injury (motor vehicle collisions, sports, and suicides) [21]. Our study also revealed a lower incidence of death in motorcycle collisions, owing to the additional protection of wearing helmets [22]. 

The assessment of overall injury severity is considered of fundamental importance in the clinical outcome of patients who suffer from STBI with extracranial trauma. In our sample, non-survivor STBI showed higher ISS and head and neck AIS scores and lower GCS and GOS, as compared to survivors. These findings are consistent with previous studies, which correlate higher AIS and ISS as well as lower GCS with worse neurological prognosis (GOS score) and STBI mortality [22,23]. We did not confirm the ability of all these scores to predict the prognosis of STBI patients in a logistic regression model. In contrast, our study confirmed that only age and admission GCS score can be used as independent predictors of mortality in STBI patients with extracranial trauma, as previous evidence has correlated [23,24]. 

In addition to clinical features, cranial CT scans play an important role in the diagnosis and prognosis of STBI. The most common CT findings in both groups were traumatic SAH (58.8%) and SDH (56.9%), followed by cerebral contusion (27.5%). Notably, non-survivors had more IPH, swelling, and radiological signs of brain herniation compared to survivors, in which cerebral contusion was more frequent. For this reason, due to the similar proportion of surgically treatable lesions in both groups, as well as the presence of a higher number of initial poor prognosis lesions that discourage surgical treatment in the dead group, no differences were found in the number of urgent neurosurgeries between groups. In this regard, previous studies have demonstrated that some initial CT findings, such as signs of brain shift and intraparenchymal hemorrhage, are more likely to have PHI [25], coagulopathy, and mortality [26,27]. 

Patients who sustained a traumatic intracranial hemorrhage and/or increased ICP at admission CT scan remain at risk for developing a coagulopathy until 72 h after trauma, showing worse clinical outcomes than patients who do not develop a coagulopathy [6]. Coagulation disorders in TBI are complex and characterized by the consumption of clotting factors and platelets, platelet dysfunction, and hyperfibrinolysis [28]. Although coagulation abnormalities have been studied previously in the isolated TBI population [6,29], this is one of the few reports showing the occurrence of a coagulopathy in STBI patients who present traumatic intracranial hemorrhage with associated extracranial injuries.

Diagnostic criteria for coagulopathy are inconsistent in the literature, but it may be recognized by conventional lab tests [30]. In our study, we observed that clotting parameters, platelets, and hemoglobin deteriorated gradually from admission until 48 h, with significant differences between groups depending on the measured time. We only found statistical differences between survivors and dead regarding PT, INR, and APTT at admission to the SICU. These abnormalities between ED arrival and SICU admission might be explained because lab tests at SICU admission are usually done some hours after the injury and ED arrival, reflecting that hemostatic function changes over time, as compensatory mechanisms are engaged and inflammation progresses [31]. Different previous studies have shown inconsistent results regarding conventional clotting tests. The incidence of an elevated PT, INR, or APTT in TBI varies widely, from 1% to 31% [32], mostly affected by testing time and injury severity. In a recent systematic review, Fletcher-Sandersjöö [29] found that most of studies noted that TBI was followed by a decrease in markers of coagulation cascade function. Furthermore, not all patients with abnormalities in laboratory coagulation tests are bleeding [9]. In fact, Juratli and coworkers [33] found no significant difference in relation to an elongated INR or APTT and PHI. Thus, PT, INR, or APTT have not been proven to be a risk factor or an exclusive indicator of PHI [30]. 

We found that the platelets count at ED admission was the only significant blood parameter in a multivariate logistic regression model, apart from age and GCS score. In our study, platelet levels significantly decreased in both groups after STBI, highlighting a lower platelet count since arrival to ED in patients who eventually died. In addition, this decrease in platelet count appeared to be independent of other hemostatic disorders (liver insufficiency, alcohol consumption, etc.) and antithrombic therapy. Different studies have previously correlated thrombocytopenia with worse outcomes in patients with TBI [8,34,35]. Our results are consistent with Carrick’s report [36], who found that patients with moderate and STBI are at risk of developing progressive thrombocytopenia after admission. To the best of our knowledge, few studies have examined the connection between thrombocytopenia and clinical outcomes in patients with non-isolated STBI. Lillemäe et al. [34] and Engström et al. [37] recently found that a decrease in platelets occurs after isolated TBI, and thrombocytopenia (<100 × 10^3^/μL platelets) during the first 24 h predicts mortality. These studies corroborate our findings regarding the association between thrombocytopenia and short-term mortality in patients with non-isolated TBI and reinforce the idea that thrombocytopenia is a potent predictor in the prognoses of STBI patients. 

The reason behind our findings is likely multifactorial. Consumption of platelet–fibrin clot formation probably early decreases platelet count, and this decrease is aggravated by dilution due to fluid resuscitation. Moreover, extracranial injuries potentially could contribute to greater consumption of platelets at an earlier time compared to isolated STBI, promoting the progression of brain bleeding. Besides platelet count, platelet dysfunction has been shown to be present after TBI, contributing also to hemorrhagic complications [38]. We were not able to measure platelet function, but it is assumed that decreased platelet counts and hematocrit may also reflect a decreased platelet function [39]. Additional decreases in platelet count and function may be the result of consumptive depletion and exhaustion [8]. Finally, platelet dysfunction could also be driven by endothelial injury [40] and structural changes following trauma, which could decrease their function [39]. 

So far, the role of platelets in trauma-induced coagulopathy remains unclear. Variations of platelet count reflect trauma severity, and several observational studies have confirmed that admission platelet count is a biomarker of trauma severity, associated with injury severity, shock intensity, and coagulopathy at admission [41]. Platelet count declines more in patients with PHI [37], more severe injuries, and older age [42]. In our study, 88% of patient deaths were due to neurological causes (brain death or devastating brain injury), indicating that most of the mortality overserved could be explained by the severity of the primary brain injury, secondarily exacerbated by a thrombocytopenia-induced PHI. However, as we did not assess subsequent cranial CT reports to demonstrate PHI, we cannot affirm if thrombocytopenia is a primary cause of mortality due to PHI or if it is secondary to other pathophysiological processes, such as cerebral hemorrhage itself raised intracranial pressure or bleeding from extracranial lesions. Even so, our findings suggest that low platelet count at ED arrival might perhaps exacerbate the primary and secondary brain injury through PHI, but also that a low platelet count at ED arrival could be an indirect marker of a more severe brain injury and, thereby, a higher mortality. In any case, what is clear is that patients with a very low platelet count are at higher risk of increased mortality, though the overall risk of death is also determined by other risk factors (age, comorbidities, and severity of extracranial injuries) and other coagulation disorders. 

Of note, we found that STBI with <150 × 10^3^/μL platelets on ED arrival had a significant early drop in survival compared to >150 × 10^3^/μL platelets patients. In the literature, the definition of thrombocytopenia varies among studies, ranging from <50 × 10^3^ platelets/μL to <150 × 10^3^ platelets/μL [43,44], but a threshold of 100 × 10^3^ platelets/μL seems to be the most frequently used definition of thrombocytopenia in trauma, and this is also the target level referred to in the European trauma guidelines for patients with TBI [15] and Brain Trauma Foundation guidelines [12]. Despite this, the quality of evidence that supports this hemostatic threshold in trauma is very low and was defined from a small series of surgical patients with isolated thrombocytopenia and not from severe trauma patients as well, as they do not consider trauma-induced platelet dysfunction. Actually, it has been described that a platelet count < 175 × 10^3^ platelets/μL is a significant predictor of PHI, while a lower count of <100 × 10^3^ platelets/μL during the first day is associated with a ninefold adjusted risk of death in isolated STBI [45]. In this respect, in our study, we not only found an association between thrombocytopenia and mortality, but our results also suggest that even a “normal” platelet count (150 × 10^3^/μL platelets) at the early time of ED arrival might not be enough to rule out the risk of death in non-isolated STBI. In fact, our findings would point out that thresholds of guidelines (100 × 10^3^ platelets/μL) may be insufficient in STBI associated with extracranial injuries and expose an additional argument to increase platelet transfusion threshold in order to prevent the initial hemorrhage to further expand and cause secondary injury.

Whether platelet transfusion can reduce mortality induced by thrombocytopenia after STBI is still a question to be answered by appropriate studies, previous data have suggested that early platelet transfusion may have an independent role in survival after TBI [46,47], while small observational trials have suggested no efficacy of platelet transfusion after TBI, despite documented thrombocytopenia or other platelet dysfunction [48,49]. Although in our study we didn’t evaluate the impact of platelet transfusion on STBI mortality, in the absence of available randomized clinical trials and given the low evidence quality that currently guides thrombocytopenia thresholds in trauma guidelines, our study prompts that platelet transfusion could be used as a potential tool for the treatment of thrombocytopenia (with a threshold of <150 × 10^3^/μL platelets) in patients with non-isolated STBI due to its low risk/reward ratio. However, in the future, prospective clinical trials should be designed as a priority to determine the beneficial effects of early treatment of thrombocytopenia through platelet transfusion.

Platelet transfusion could prevent the development of PHI, but not the brain damage caused by the initial bleeding. Intracranial bleeding triggers oxidative stress and free radical formation, enhancing the inflammatory response that exacerbates secondary injury, and may induce PHI. In this sense, treatments that can recover injured brain tissue after initial bleeding and PHI are currently lacking. After STBI, blood-brain barrier integrity is altered, which may provide a window of opportunity to deliver drugs effectively into the haematoma and surrounding injured brain tissue, such as antioxidant nanoparticles (ANPs) [50]. ANPs have shown promising outcomes in reducing the progression of TBI when administered immediately following an injury. ANPs have antioxidant and anti-inflammatory properties, which may improve STBI outcomes by reducing the secondary injury of TBI through haematoma and oedema resolution and enhacing neuroplastic and neurogenesis abilities of the brain [51]. Therefore, these materials represent a novel improved treatment strategy to reduce the secondary spread of TBI.

Regarding fibrinogen levels, they raised in both groups at 24 h after SICU admission, when non-survivors showed significant lower fibrinogen levels compared to survivors. This result may suggest an underlying hyperfibrinolysis mechanism in non-survivors, which has previously been associated with early mortality due to increased bleeding, while survivors would experience hypofibrinolysis or fibrinolysis shutdown, which modifies coagulopathy toward a less coagulopathic state [52].

Finally, an aggressive hemostatic resuscitation and pre-hospital fluid administration could worsen TBI-related coagulopathy by dilution, acidosis, and hypothermia [34]. In our sample, there were no differences between survivors and non-survivors with respect to pre-hospital fluid replacement, time to hospital admission, hemodynamics, RBC transfusion, or need for activation of MTP. Actually, STBI survivors had higher levels of hemoglobin within the first 24 h after admission despite a higher rate of urgent surgery and neurosurgery, suggesting that an endogenous “early hypocoagulability state” would develop in some patients after STBI since no differences in transfusion and pre-hospital fluids were observed, which would mainly affect the iatrogenic exogenous coagulopathy before and shortly after admission. The lower rates of urgent surgery and neurosurgery in the dead group may be explained by the presence of more serious injuries, with worse vital prognosis.

In view of previous, our results underline that age, GCS, and thrombocytopenia at ED arrival adversely affect polytrauma with STBI outcomes. In recent years, significant progress has been made in understanding STBI coagulopathy, but gaps remain, as discussed above. In our study, early thrombocytopenia predicts mortality in non-isolated STBI. Due to the retrospective characteristics of our study and the lack of follow-up radiological data, we do not presently know whether the initial low platelet count observed is a causal factor of PHI or a coexisting marker of greater brain injury. Despite this, our findings emphasize the importance of identifying STBI patients with <150 × 10^3^ platelets/μL at ED arrival since they are likely at a higher risk of death. Therefore, we consider that our results are a very interesting finding since alert clinicians from the ED arrival. Although this type of patient has a level of platelets that we a priori consider safe, in reality, it may not be serving as a trigger to assess platelet count at ED arrival, closely monitor (performing earlier control CT scans after admission, closer neurological surveillance, etc.), and early platelets transfuse. Finally, since our findings provide a rationale for future studies of PHI and hemostatic agents in the treatment of TBI, preferably prospective clinical trials with serial imaging and clinical follow-up after discharge should be designed as a priority to determine the beneficial effects of early treatment of thrombocytopenia in order to establish causal mechanisms between clinical management, thrombocytopenia, PHI, and mortality associated to STBI.

### Limitations

Our study has some limitations due to its retrospective design. First, we were unable to obtain more precise hemostatic lab data regarding platelet function, thromboelastography, or clotting factor levels in order to better characterize the mechanisms underlying STBI-induced coagulopathy. However, these tests are labor-intensive and require specialized equipment not available at all centers in the ED, which makes their application in the clinical setting difficult. Moreover, we did not have access to follow-up radiological data on intracranial hemorrhage progression. For this reason, we could not confirm a simple causal relationship between a low platelet count and PHI, which is also influenced by direct injury to blood vessels and inflammation caused by initial primary injury. Finally, we were unable to record differences in platelet transfusions between groups, although there is still a lack of clear evidence of mortality benefit of platelet transfusion in patients with TBI. Despite its limitations, we believe that clinically helpful and valid conclusions can be drawn from this research.

## 5. Conclusions

Older age, GCS score, and platelet count at ED arrival are independently associated with higher mortality in STBI patients with extracranial trauma. Thrombocytopenia < 150 × 10^3^ platelets/μL at ED arrival is a simple prognostic tool to early predict survival or death between non-isolated STBI patients. Clinical trials are necessary to identify if platelet transfusions aiming to avoid early thrombocytopenia would improve outcomes in these patients.

## Figures and Tables

**Table 1:** Epidemiological and clinical characteristics of non-isolated STBI patients.

	Total (*n* = 134) Median (IQR)	Survivors (*n* = 83) Median (IQR)	Dead (*n* = 51) Median (IQR)	*p*
Age (years)	45 (30–65)	39 (29–55)	57 (36–78)	0.003
Male sex, *n* (%)	86 (64.1%)	53 (63.8%)	33 (64.7%)	0.981
Time taken to reach hospital (min)	51 (41–61)	52 (43–59)	49 (40–65)	0.508
Mechanism of trauma				
Car, *n* (%)	13 (9.7%)	9 (10.8%)	4 (7.8%)	0.608
Motorcycle, *n* (%)	28 (20.9%)	22 (26.5%)	6 (11.7%)	0.039
Knocked down, *n* (%)	43 (32.1%)	25 (30.1%)	18 (35.3%)	0.454
Fall, *n* (%)	50 (37.3%)	27 (32.5%)	23 (45.1%)	0.387
Pre-hospital fluid replacement (mL)	600 (300–1200)	650 (300–1275)	600 (200–1100)	0.571
Systolic arterial pressure (mm Hg)	120 (90–140)	119 (90–136)	120 (81–150)	0.916
Heart rate (bpm)	97 (79.75–120.5)	100 (82–120)	90 (79–124)	0.369
Glasgow Score	6.5 (3–13)	9 (4–13)	4 (3–7)	<0.001
AIS, head and neck	4 (3–5)	4 (3–4)	5 (4–5)	<0.001
AIS, overall	10 (7–12)	9 (8–11)	10 (5–13)	0.897
ISS	34 (29–43)	34 (27–41)	41 (34–50)	0.003
Length of stay in SICU (days)	9 (3–25.25)	17 (8–32)	2 (0–5)	<0.001
Red blood cell units, *n*	0 (0–5)	0 (0–4)	0 (0–6.75)	0.638
MTP, *n* (%)	28 (20.7%)	17 (20.2%)	11 (21.6%)	0.853
GOS	3 (1–5)	3 (2–5)	1 (1–1)	<0.001
Need for urgent surgery, *n* (%)	77 (57%)	59 (70%)	18 (35%)	<0.001
Need of urgent neurosurgery, *n* (%)	36 (26.8%)	24 (28.9%)	12 (23.5%)	0.52

Categorical data are shown in sample size (*n*) and percentages. Quantitative data are shown as median (percentile 25–percentile 75) as not normally distributed. Abbreviations: *n*, number; IQR, interquartile range; min, minutes; mL, milliliters; bpm, beats per minute; AIS, Abbreviated Injury Scale; ISS, Injury Severity Score; MTP, massive transfusion protocol; GOS: Glasgow Outcome Score.

**Table 2:** Neuroradiological findings in admission CT.

	Survivors (*n* = 83) *n*, (%)	Dead (*n* = 51) *n*, (%)	*p*
SAH	47 (56%)	30 (58.8%)	0.744
SDH	35 (41.7%)	29 (56.9%)	0.086
EDH	9 (10.7%)	1 (2%)	0.06
IPH	2 (2.4%)	12 (23.5%)	<0.001
IVH	7 (8.3%)	5 (9.8%)	0.771
Cerebral contusion	30 (35.7%)	14 (27.5%)	0.034
Brain stem hemorrhage	1 (1.2%)	2 (3.9%)	0.319
Brain swelling	3 (3.6%)	7 (13.7%)	0.211
Brain herniation sings	6 (7.1%)	12 (23.5%)	0.007
Skull fracture	34 (40.5%)	28 (54.9%)	0.103

Categorical data are shown in sample size and percentages. Abbreviations: *n*, number; SAH, subarachnoid hemorrhage; SDH, subdural hemorrhage; EDH, epidural hemorrhage; IPH, intraparenchymal hemorrhage; IVH, intraventricular hemorrhage.

**Table 3:** Associated extracranial injury of non-isolated STBI patients.

	Total *n*, (%)	Survivors *n*, (%)	Dead *n*, (%)	*p*
Face	41 (30.6%)	23 (27.7%)	18 (35.3%)	0.335
Spine	63 (47%)	42 (50.6%)	21 (41.2%)	0.288
Mediastinum	9 (6.7%)	6 (7.2%)	3 (5.6%)	0.762
Thorax	89 (66.4%)	60 (72.2%)	29 (56.8%)	0.066
Abdomen	46 (34.3%)	30 (36.1%)	16 (31.3%)	0.572
Pelvic fracture	42 (31.3%)	25 (30.1%)	17 (33.3%)	0.697
Skeleton	88 (65.6%)	55 (66.2%)	33 (64.7%)	0.854
Liver	22 (28.2%)	17 (20.5%)	5 (9.8%)	0.167
Spleen	9 (13.2%)	7 (8.4%)	2 (3.9%)	0.289
Massive hemoperitoneum	1 (1.5%)	1 (1.2%)	0 (0%)	0.426
Diaphragm	1 (1.5%)	1 (1.2%)	0 (0%)	0.426
Kidney	11 (15.7%)	7 (8.4%)	4 (7.8%)	0.87
Retroperitoneal hematoma	22 (29.7%)	13 (15.6%)	9 (17.6%)	0.723

Categorical data are shown in sample size and percentages. Abbreviations: *n*, number.

**Table 4:** Laboratory results for clotting parameters, hemoglobin, and platelet count since admission to 48 h in the SICU.

		ED Arrival		SICU Admission		24 h at SICU		48 h at SICU	
		Median (IQR)	*p*	Median (IQR)	*p*	Median (IQR)	*p*	Median (IQR)	*p*
**INR** **(s)**	**Survivors**	1.07 (1.00–1.3)	0.203	1.11 (1.02–1.28)	0.01	1.16 (1.06–1.30)	0.287	1.10 (1.02–1.22)	0.173
	**Dead**	1.18 (1.01–1.48)		1.19 (1.08–1.54)		1.19 (1.08–1.42)		1.11 (1.04–1.43)	
**APTT** **(s)**	**Survivors**	28.8 (26.5–34.4)	0.167	28.5 (26.2–32.9)	0.04	29.1 (27.4–31.1)	0.584	29.0 (27.3–31.0)	0.371
	**Dead**	32.9 (25.8–45.6)		34.0 (26.4–43.9)		29.7 (26.3–33.7)		29.7 (26.6–33.3)	
**PT** **(s)**	**Survived**	12.8 (11.8–15.5)	0.184	13.2 (12.1–15.5)	0.006	13.9 (12.6–15.6)	0.287	13.2 (12.0–14.6)	0.514
	**Died**	14.1 (12.0–17.8)		14.3 (12.9–18.7)		14.2 (12.8–17.1)		13.2 (12.3–16.8)	
**Fib** **(g/L)**	**Survivors**	12.8 (11.8–15.5)	0.184	13.2 (12.1–15.5)	0.006	13.9 (12.6–15.6)	0.287	13.2 (12.0–14.6)	0.277
	**Dead**	14.1 (12.0–17.8)		14.3 (12.9–18.7)		14.2 (12.8–17.1)		13.2 (12.3–16.8)	
**Hb** **(g/dL)**	**Survivors**	12.4 (10.5–13.8)	0.01	11.8 (10.4–13.2)	0.001	10.2 (9.2–11.6)	0.238	9.1 (8.2–10.4)	0.927
	**Dead**	10.5 (7.8–13.2)		10.1 (8.6–12.1)		9.8 (8.7–11.3)		9.5 (8.4–10.4)	
**PC** **(×10^3^/L)**	**Survivors**	200 (159–238)	<0.001	192 (134–235)	<0.001	143 (108–176)	<0.001	124 (100–164)	0.001
	**Dead**	149 (114–184)		130 (98–160)		107 (81–129)		92 (70–130)	

Quantitative data are shown as median (percentile 25–percentile 75) as not normally distributed. Abbreviations: IQR, interquartile range; PT, platelets; INR, International Normalized Ratio; APTT, activated partial prothrombin time; PT, prothrombin time; Fib, fibrinogen; Hb, hemoglobin; PC, platelet count; ED, emergency department; SICU, surgical intensive care unit.

**Table 5:** Statistical results of the logistic regression analysis for mortality.

	B	S.E.	Wald	df	Sig.	Exp(B)
Age	0.037	0.011	11.352	1	0.001	1.037
Glasgow Coma Scale score	−0.226	0.055	16.914	1	<0.001	0.797
Platelet count on ED admission	−0.01	0.003	8.976	1	0.003	0.99
Constant	1.15	0.847	1.843	1	0.175	3.159

Abbreviations: B, estimated parameter; S.E, standard error; df, degrees of freedom; sig., signification; Exp(B), B exponential; ED: emergency department.

## Data Availability

The data presented in this study are available on request from the corresponding author due to privacy and ethical restrictions.
